# Abnormal origin of the left pulmonary artery

**DOI:** 10.1093/ehjcr/ytae280

**Published:** 2024-06-11

**Authors:** Chunyi Zhou, Yurong Guo, Yancong Zeng, Honghong Ke

**Affiliations:** Department of Radiology, The First Affiliated Hospital of Guangxi Medical University, No.6 Shuangyong road, Nanning, Guangxi 530021, China; Department of Radiology, The First Affiliated Hospital of Guangxi Medical University, No.6 Shuangyong road, Nanning, Guangxi 530021, China; Department of Radiology, The First Affiliated Hospital of Guangxi Medical University, No.6 Shuangyong road, Nanning, Guangxi 530021, China; Department of Geriatric Cardiology, The First Affiliated Hospital of Guangxi Medical University, No.6 Shuangyong road, Nanning, Guangxi 530021, China

## Case description

**Figure 1:**
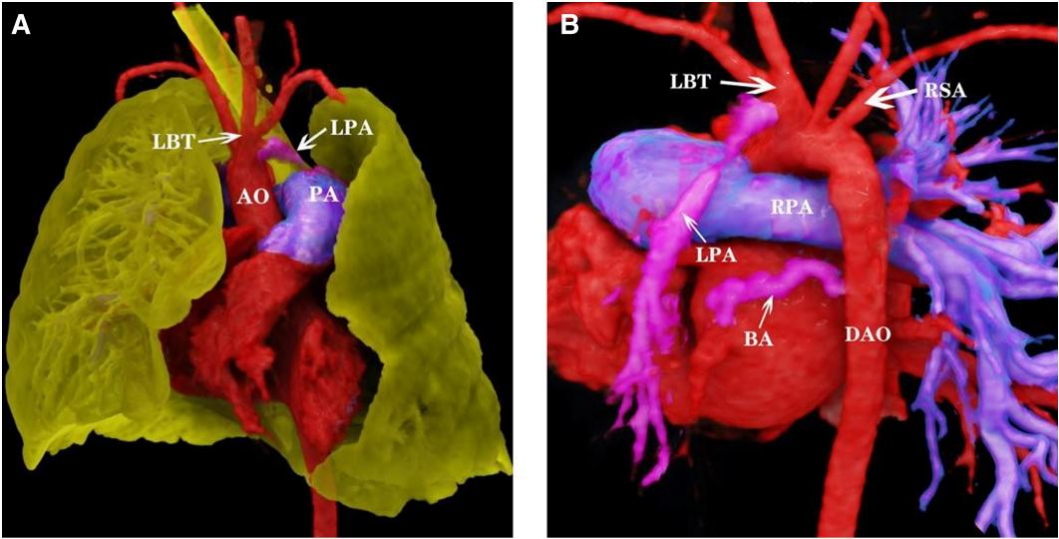
A 1-month-old boy’s left pulmonary artery heterotopia originated from computed tomography angiography of the left brachiocephalic trunk. (*A*, *B*) Cinematic rendering images showed that the left pulmonary artery heterotopic originated from the left brachiocephalic trunk. AO, ascending aorta; BA, bronchial artery; DAO, descending aorta; LBT, left brachiocephalic trunk; LPA, left pulmonary artery; PA, pulmonary artery; RPA, right pulmonary artery; RSA, right subclavian artery.

Anomalous origin of pulmonary artery (AOPA) is a rare congenital heart anomaly characterized by the main pulmonary artery entering one lung hilus through a blood vessel, while the other lung is supplied by abnormal blood vessels directly originating from the aorta (*Figure 1A* and *B*). The prevalence of AOPA in patients with congenital heart disease is 0.33%,^1^ with left pulmonary artery abnormalities originating from the aorta, accounting for approximately 25% of cases; the abnormality of the right pulmonary artery originates from the aorta, accounting for approximately 75% of cases; it is extremely rare that the abnormal left pulmonary artery originates from the left brachiocephalic trunk. Computed tomography angiography helps in providing exact anatomical delineation and identifying associated anomalies, thus aiding pre-operative planning of surgical management.^2^

**Consent:** The authors confirm that written consent for submission and publication of this case report including images and text has been obtained from the patient in line with COPE guidance.

**Funding:** None declared.

## Supplementary Material

ytae280_Supplementary_Data

## Data Availability

The data underlying this article are available in the article and in its online Supplementary material.
