# Supplementary figures and images for: Abnormal origin of the left pulmonary artery
Source: Eur Heart J Case Rep. 2024 Jun 11;8(6):ytae280. doi: 10.1093/ehjcr/ytae280 (PMC11211917; doi:10.1093/ehjcr/ytae280)

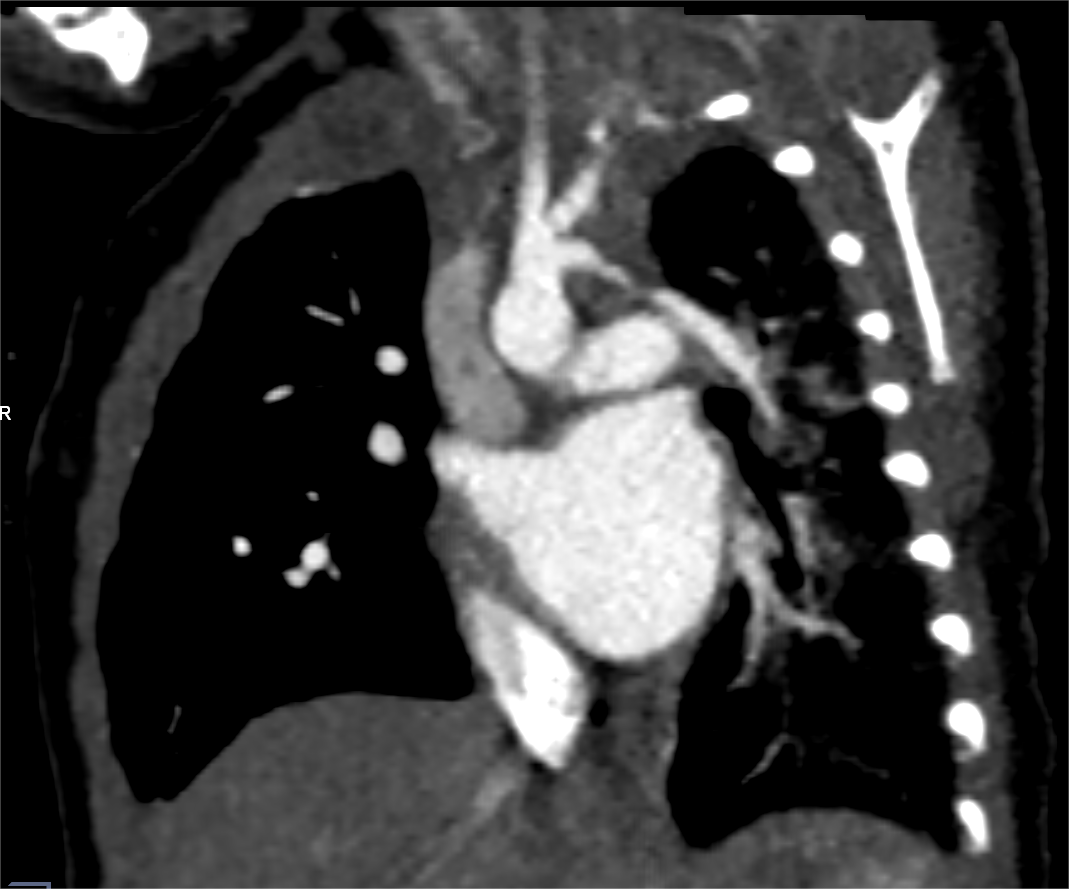

Supplement: ytae280_Supplementary_Data [file ytae280_supplementary_data.zip › Supplementary file-Figure.tif]
